# From Cognitive Function to Treatment Efficacy in Obsessive–Compulsive Disorder: Insights from a Multidimensional Meta-Analytic Approach

**DOI:** 10.3390/jcm13164629

**Published:** 2024-08-07

**Authors:** Ben Harkin, Alan Yates

**Affiliations:** Department of Psychology, Manchester Metropolitan University, All Saints Building, Manchester M15 6BH, UK; a.yates@mmu.ac.uk

**Keywords:** obsessive–compulsive disorder, memory performance, multidimensional meta-analysis (mi-MA), complex cognitive models, therapeutic targets

## Abstract

Meta-analysis is a statistical tool used to combine and synthesise the results of multiple independent studies on a particular topic. To this end, researchers isolate important moderators and mediators to investigate their influence on outcomes. This paper introduces a novel approach to meta-analysis, known as multidimensional meta-analysis (mi-MA), to study memory performance in those with obsessive–compulsive disorder (OCD). Unlike traditional meta-analyses, mi-MA allows researchers to extract multiple data points (e.g., using different measures) from single studies and groups of participants, facilitating the exploration of relationships between various moderators while avoiding multicollinearity issues. Therefore, in the first instance, we outline the use of the mi-MA approach to quantify the impact of complex models of memory performance in individuals with OCD. This approach provides novel insights into the complex relationship between various factors affecting memory in people with OCD. By showcasing the effectiveness of mi-MA in analysing intricate data and modelling complex phenomena, the paper establishes it as a valuable tool for researchers exploring multifaceted phenomena, both within OCD research and beyond.

## 1. Introduction

Obsessive–compulsive disorder (OCD) is a debilitating mental health condition characterised by intrusive thoughts (obsessions) and repetitive behaviours (compulsions), according to the APA [1]. Its impact extends widely, with approximately 520,000 individuals experiencing clinical manifestations and around 7,000,000 exhibiting subclinical symptoms in the United Kingdom (Sunol et al. [2]). Notably, OCD has significant consequences, including a three-fold increase in suicide risk (Cruz et al. [3]), a burden on family members comparable to that of schizophrenia [4], and a ten-fold-higher likelihood of unemployment (Rodriguez-Salgado et al. [5]). Furthermore, it is linked to various cognitive impairments [6], prompting inquiries into multiple cognitive domains to enhance our understanding of the disorder (for reviews, see [7,8,9,10,11,12,13,14,15,16,17,18]) and develop treatments for it, e.g., [19,20,21,22,23,24,25,26,27,28,29]. One particularly fruitful area of research is the role of memory in the development and maintenance of OCD [7,30,31,32]. As early as 1977, Reed identified that “perhaps the most central feature of obsessional disorder seems to involve pathologically faulty memory” [33], which sparked a growing interest in this area of research thereafter, e.g., [34,35,36].

Despite substantial advancements in comprehending the role of memory in OCD (for excellent reviews, see [7,15,16,17]), a unified and coherent explanation has proven difficult to achieve [37]. Recent multidimensional meta-analyses (mi-MAs) conducted by Perrson et al. [38] and Harkin et al. (2023) (see also [39,40]) have attempted to bridge this gap. Specifically, the mi-MA approach facilitates the exploration of intricate and multifaceted models within OCD and other areas of psychology, e.g., [41]. Unlike traditional approaches that merely compare effect size differences between moderators, mi-MA enables researchers to delve deeper into factors that contribute to the most significant variability in outcomes. Moreover, this approach accommodates the interdependence of effect sizes, as highlighted by Viechtbauer [42], by allowing for the extraction of multiple data points from the same study and group of participants.

To achieve this, the present paper employs the following structure:

(a) We first review the existing body of research on memory performance within the realm of OCD. Following this, we introduce a pivotal concept—the EBL classification system [43]—which combines executive function (E), binding complexity (B), and memory load (L) to provide a framework for examining memory impairments in OCD. We highlight its empirical basis and its ability to address various task requirements in elucidating the occurrence, absence, and extent of memory deficits in individuals with OCD. However, while EBL provides valuable insights, we also recognise its limitations, including its theoretical framework and the difficulty in quantifying its dimensions.
(b) To address these limitations, we then elucidate how we used the mi-MA approach [38] to quantify the impact of task demands in terms of E, B, and L dimensions on the memory performance of those with OCD. We contend that this offers a more thorough comprehension of how these factors interact with memory performance in OCD than was previously achievable.
(c) We then delve into our refined analysis of specific facets of executive control [39], analysing the impacts of top-down and bottom-up processing, and their individual roles in memory performance in OCD, we underscore how our mi-MA approach enabled us to measure these processes in OCD, a novel approach not previously explored in the literature.
(d) As our paper nears its conclusion, we reiterate the significance of our findings and underscore the importance of research that addresses the clinical relevance of cognitive factors in OCD. By leveraging the wealth of existing research and integrating their findings through an mi-MA lens, we then propose novel insights into the cognitive processes of OCD and inform the development of more effective interventions. We hope that our approach serves to address Ouimet et al.’s [44] critique of cognitive research in OCD, which states that “simply explaining the cognitive phenomenology of OCD without a direct view towards enhancing its clinical relevance, although interesting, is unlikely to be helpful” (p. 26).

## 2. Memory Performance in OCD: Insights from Mixed Findings and the EBL Classification System

As touched upon previously, memory performance in OCD is well researched, with numerous aspects of memory associated with the maintenance and development of OC symptomatology [21,32,45,46,47,48,49,50,51,52]. However, despite this extensive research, the evidence regarding memory performance in individuals with OCD presents a mixed picture at best [37]. For example, some studies report a memory [28] while others yield conflicting results [53,54]. Similarly, studies have demonstrated both impaired verbal memory [55] and intact performance in this domain [56]. Visuospatial memory has also been found to be generally affected in some studies [16,37,50] while intact in others [56,57]. Thus, as we previously suggested, a unified and coherent approach to understanding memory performance in OCD is lacking.

The initial step towards a solution was proposed by Greisberg and McKay [21], who suggested a more subtle approach to understanding memory performance in individuals with OCD, noting that memory impairments are secondary to executive dysfunction. As Harkin and Kessler [43] went on to elucidate, “If a memory task taps into a dysfunctional component of executive functioning, memory impairment will follow” (p. 1005). In essence, it is the cognitive demands imposed by a particular memory task on executive functioning that distinguish the memory performance of individuals with OCD from that of control subjects [17]. Building upon the foundational executive memory explanation, Harkin and Kessler [43] further expanded the framework by incorporating three essential components of a task: executive functioning (E), binding complexity (B), and memory load (L), which resulted in the development of the EBL classification system. Executive function was defined after Diamond’s [58,59] tripartite definition, consisting of inhibition (e.g., top-down selective activation of task-relevant representations and inhibition of task-irrelevant stimuli and responses), working memory (maintenance and updating), and flexibility (the capacity to dynamically distribute resources for processing and managing information in an ever-evolving environment) (see also [60]). Binding complexity refers to the extent that a WM task requires the binding of multimodal conjunctions in the pursuit of successful task performance [61]. Load refers to the number and amount of information that enters the WM either concurrently or sequentially [62].

The EBL classification system was developed based on a series of interconnected empirical foundations. It utilised the robust empirical basis of Baddeley’s model of working memory (WM), specifically the role of binding within the episodic buffer [63,64]. Accurate memory involving both WM and long-term memory (LTM) requires the encoding, maintenance, and retrieval of connections among various elements within a multimodal experience [65]. To address this, Baddeley [63] expanded his well-known 1986 WM model [66] to include an episodic buffer, which allowed for the temporary integration of multimodal representations and served as a gateway to episodic LTM. Building on this, Harkin and Kessler [67] proposed that executive dysfunction (e.g., uncontrolled intrusive thoughts/stimuli) in individuals with OCD disrupts fragile multimodal bindings within the episodic buffer, leading to the impaired consolidation of episodes in both WM and LTM.

To investigate this, Harkin and colleagues conducted a series of studies showing that individuals with OCD symptoms had impaired memory performance when tasks required sustained bindings in the episodic buffer and triggered specific aspects of their executive dysfunctions [43,68,69,70]. Specifically, they undertook WM tasks that engaged the episodic buffer by using stimuli that necessarily required the binding of multimodal conjunctions between various object features and spatial locations. Then, they interfered with episodic buffer functionality by presenting misleading/irresolvable information during the WM retention period that those with OCD would find difficult to ignore. They consistently observed that those with OC symptoms suffered attenuated performance when presented with misleading/irresolvable information but not when it was relevant/resolvable or absent (i.e., a measure of WM capacity). This later point concurs with the findings in [14,56,57] indicating that individuals with OCD do not experience impairment in working memory capacity itself.

By confirming and extending the original supposition of Greisberg and McKay [21], Harkin and colleagues emphasised that memory impairment in individuals with OCD is secondary to executive dysfunction, with the degree of impairment determined by the specific task demands. These studies played a crucial role in informing the executive function (E) and binding complexity (B) aspects of the EBL system and highlighting their significance in understanding memory impairments in OCD. Moreover, we incorporated load (L) into the EBL taxonomy, taking into account the body of research that revealed impairments in WM performance in individuals with OCD when the memory load was high but not low [71,72]. For example, the importance of load was further underscored by the meta-analysis conducted by Snyder et al. [73] who reported that those with OCD exhibited the largest magnitude of impairment on the n-back task (at high loads) compared to other tests of visual and verbal memory.

**Figure 1:**
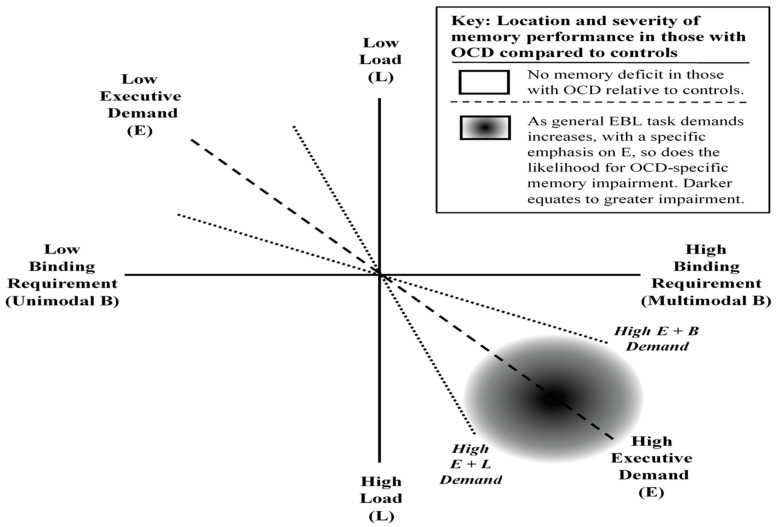
The executive function, binding complexity and memory load (EBL) classification system, adapted from Harkin and Kessler [43]. ***Notes.*** It is essential to understand that Harkin and Kessler’s three EBL dimensions are not entirely orthogonal in practical experimental scenarios and are only used as theoretical constructs. Binding complexity can impact memory load when numerous multimodal features need to be integrated. Notably, complex bindings and increased load tap into executive functions when limits are surpassed (refer to E + L and E+B oblique dimensions in the figure). Hence, we suggest that executive demands are the most crucial dimension (Harkin and Kessler 2011 [43]). The grey-scaled circular area illustrates a higher probability of an OCD memory deficit under conditions of high load and binding complexity, particularly when executive demands rise (darker areas indicate higher likelihood). Thus, the orthogonal dimensions are abstract constructs, whereas the oblique dimensions (indicated by dashed lines) depict real-world relationships: (a) the probable interrelationship between EBL factors and (b) the importance of executive function across binding complexity, memory load, and overall memory performance in OCD.

Thus, the EBL classification system emerged to explain where memory impairments would and would not occur in those with OCD (see Figure 1). This approach shifted the emphasis from focusing on general amnestic performance or domains—i.e., visual versus verbal memory—to looking at the specific content and demands of the tasks where those with OCD have intact versus impaired performance. This enabled us to explain phenomena such as unaltered and compromised verbal memory, e.g., intact: Henseler et al. (2008) [56] vs. deficit: [55,56], as well as unaltered and compromised visual memory, e.g., intact: Henseler et al. (2008) [56] vs. deficit: [16,37,50,56], across a range of differing tasks, and generally intact WM capacity [17,74,75,76].

## 3. Quantifying the EBL Classification System through a Multidimensional Meta-Analysis

A clear drawback of the initial EBL classification system of Harkin and Kessler [43] was its theoretical nature. It relied on qualitative inferences to establish the connection between task demands along the EBL dimensions and memory performance in individuals with OCD, thus lacking a quantitative foundation. As such, the next logical step was to conduct a meta-analysis in which we standardised each dimension of the EBL taxonomy, coded the included studies for each dimension, and quantified how they influenced memory performance in individuals with OCD. To establish a quantitative foundation for the EBL classification system, our meta-analysis, based on the work conducted by Persson, Yates, Kessler, and Harkin [38], delved into the influence of task demands along the EBL dimensions on memory performance in individuals with OCD. By systematically analysing a wide range of studies, we aimed to understand the intricate interplay between task demands and memory performance, shedding light on the underlying mechanisms of memory impairments in OCD.

While a full exposition of the methods employed in our multidimensional meta-analytical approach is beyond the remit of this paper, a full exposition can be found in the paper and supplimentary materials of [38]; a brief overview of some of its fundamental aspects will help inform the reader. To this end, we provide a summary on why it was carried out in this way and what was undertaken, and provide insight into why an mi-MA approach was considered more optimal than a classic moderator approach. In addition, we have provided an overview of the main stages of an mi-MA in Table 1. As you will note, they bear great similarity with respect to a traditional meta-analysis; however, the mi-MA approach provides greater flexibility in the models that can be tested and the amount of data that can be extracted from each paper.

The traditional approach to meta-analysis involves scrutinizing hypotheses of interest to ascertain whether variations in effect sizes stem from specific factors. Subsequently, the goal is to determine if these effect sizes significantly diverge among two or more of these factors [77,78]. For comprehensive insights into the stages and methodologies of this form of meta-analysis, refer to [77,79]. For example, in a meta-analysis of intervention studies, it is common to compare if effect sizes differ between the intervention and control groups [80]. Typically, the procedure entails employing a process that utilises some form of sample weighting, incorporating contributions from the variance of the distribution [81]. This is then followed by pooling, and aggregating the effect sizes across the studies included, yielding an overarching estimate of the clinical effectiveness of the intervention.

**Table 1:** Overview of the main stages of the multidimensional meta-analysis (mi-MA), as utilised in the studies of Persson et al. (2021) [38] and Harkin et al. (2023) [39].

**Figure 2:**
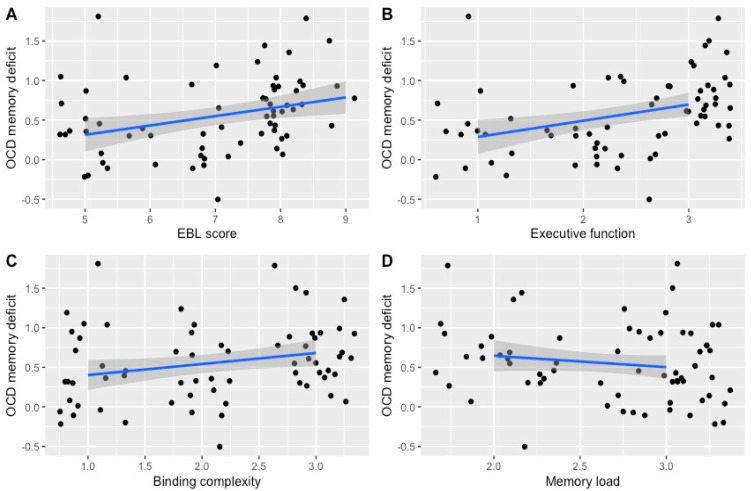
Visualisation of the individual moderator effects for total EBL model, executive function, binding complexity and memory load, as originally presented in Persson et al. [38]. We can see that memory impairment increases with increasing demands across total EBL score (**A**), executive function (**B**) and binding complexity (**C**), the converse true for the memory load (**D**). ***Notes***. The four images above depict the extent of memory deficits observed in individuals with OCD across various metrics: total EBL score (**A**), executive function (**B**), binding complexity (**C**), and memory load (**D**). The data indicate that as EBL score, demands on executive function, and binding complexity of memory tasks increase, so does the memory impairment in individuals with OCD. Conversely, higher memory load is linked with less impairment in memory for those with OCD.

**Figure 3:**
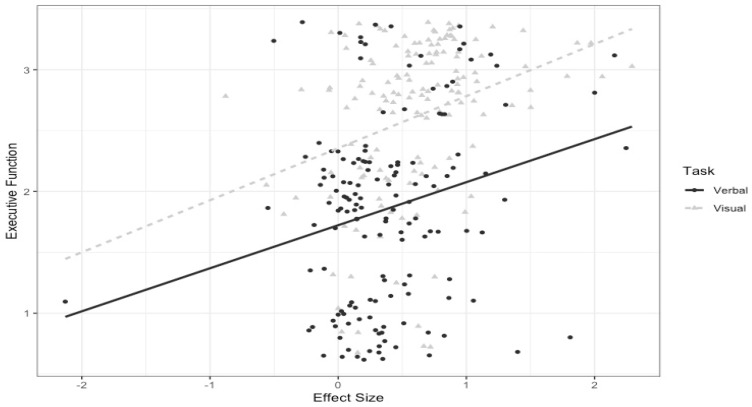
Moderating effect of executive function and task type on OCD memory deficit. The graph shows that memory impairment is more pronounced on visual tasks compared to verbal tasks, particularly when the tasks place a higher demand on executive function [38]. ***Notes***. This image illustrates the relationship between task executive function demands and the magnitude of memory impairment in individuals with OCD, expressed as an effect size (0 = no memory impairment; +2 = high memory impairment) for verbal (solid line) and visual (dashed line) tasks. It reflects the general trend observed in Figure 2B: as executive function demands increase, memory impairment in individuals with OCD also increases. Additionally, the greater memory impairment on visual tasks compared to verbal tasks is attributed to the higher executive function demands of visual tasks.

**Figure 4:**
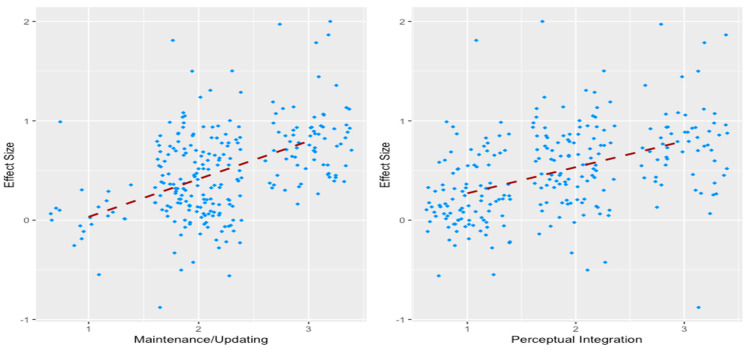
Framework moderation plots for significant moderators [39]. ***Notes.*** The left and right images depict the magnitude (effect size) of memory impairment in individuals with OCD for maintenance/updating and perceptual integration, respectively. As the demands within a task for maintenance/updating and perceptual integration increase, the memory impairment of individuals with OCD similarly increases.

Main Steps for Conducting a Multidimensional Meta-Analysis
**Define Research Questions and Variables:** Define research question and identify key variables of interest, such as cognitive (e.g., central executive, inhibition, binding), behavioural (e.g., stop-signal reaction time task, symptom severity), and neuroanatomical measures (e.g., GM volume reductions). Identify the relevant model to examine, as the mi-MA approach provides flexibility to investigate models where variables likely influence each other, e.g., executive functioning, binding complexity, memory load.Create relevant search terms to match these variables. **Systematic Literature Review:** Conduct a comprehensive search for relevant studies that include the variables of interest using databases such as Web of Science, PsycINFO, Medline, PubMed, OVID, CINAHL, PsychArticles, and ProQuest Theses. Ensure studies meet inclusion criteria for the meta-analysis. **Quantification, Data Extraction, and Coding:** Quantify and standardise the independent (e.g., E, B, L) and dependent variables (e.g., memory performance) by constructing a scoring system that is simple and produces meaningful ordinal differences for each of the independent variables. For instance, in the studies by Persson et al. (2021) [38] and Harkin et al. (2023) [39], tasks were rated on a scale from 1 to 3 based on the level of demand placed on each component: 1 signified low to minimal demand, 2 indicated moderate demands, and 3 indicated high demand. Code individual variables according to this scoring system. Extract effect sizes (e.g., Cohen’s d) for the identified dependent variables. Code additional relevant variables (e.g., sample size, demographics, comorbidities, study quality). To ensure reliability, use an independent rater for second coding. **Addressing Dependency of Effect Sizes and Statistical Analysis:** Unlike traditional meta-analyses, mi-MA enables the extraction of multiple data points from single studies and groups of participants, facilitating the exploration of relationships between various moderators while avoiding issues of multicollinearity. As such, mi-MA accounts for within-study and between-study variance. Conduct multidimensional meta-analyses using the rma.mv function in the Metafor package for R, following recommendations of Viechtbauer (2010) [42]. Apply a three-level meta-analytical framework when correlations between effect sizes are unknown. This method considers three variance components among effect sizes within the same study: variance between participants (level 1), outcomes (level 2), and studies (level 3) (Assink & Wibbelink, 2016) [82]. Consider using techniques like robust variance estimation (RVE) when handling dependent effect sizes. Calculate overall effect sizes using suitable meta-analytic models, such as the mixed-effects model, in R. Following recommendations from Hox (2010) [83] and Assink and Wibbelink (2016) [82], first examine moderators individually (E vs. B vs. L) and then in a combined analysis (EBL). This approach allows for initial significance screening while considering the potential intercorrelation of variables, which may lead to multicollinearity in analyses. By combining the variables, we can test which variable(s) account for the most variance in the model compared to the others. **Interpretation and Synthesis:** Interpret the findings in the context of the research questions. Discuss the implications for theory and practice, highlighting the transdiagnostic nature of the findings if relevant. **Reporting and Visualisation:** Utilise visual aids such as tables, forest plots, and path diagrams to depict significant findings and associations. For instance, in our meta-analysis, we visually represented our data (refer to Figure 2, Figure 3 and Figure 4 in this paper). This allowed readers to observe the three different levels (1 = low, 2 = medium, 3 = high) for each of our independent variables (e.g., EBL, E, B, L), the distribution of effect sizes (i.e., memory performance) across these levels, and the corresponding line of best fit. Present a clear, concise summary of the results and their implications.

However, this approach has certain limitations. Firstly, studies often report multiple effect sizes because participants may complete various tasks and/or measures during the study period, violating the usual requirement for independent effect size measures in meta-analysis [84,85,86]. Dependency of effect sizes typically means that effect sizes within studies are correlated, creating overlap and inflation of the data, which can lead to overconfidence in the results [82,87]. While conducting subgroup analysis or aggregating effect sizes is possible, this reduces the number of effect sizes analysed, thus limiting the power of the analysis. Additionally, this method was unsuitable for testing our model, which involves multiple factors that may interact, and required us to perform multiple moderation analyses [82].

Meta-regression also plays an important role in meta-analysis, accounting for the covariate effects of sample means or effect sizes presented as predictor or moderator variables, particularly when categorical variables are used for subgroup analysis. Meta-regression is a rigorous statistical framework valued as a sustainable approach to uncover predictors of heterogeneity in the prevalence rates of a phenomenon occurring across multiple studies [77,78,79]. A limitation of meta-regression in comparison to the mi-MA is that meta-regression typically handles covariates and moderator variables independently, which may not adequately capture the complex interactions between multiple factors influencing the outcomes. Meta-regression generally focuses on assessing how individual predictors (moderators) impact the effect sizes across studies, often treating each predictor separately or in simple combinations [88,89]. In contrast, a multidimensional meta-analysis can more effectively account for the simultaneous and interactive effects of multiple dimensions or factors. This approach is particularly advantageous in complex research areas where outcomes are influenced by several interacting variables, as it allows for a more nuanced and comprehensive understanding of how these variables jointly contribute to observed effects [84,87]. In the context of the EBL classification system, the multidimensional approach enables the examination of the combined and interactive influences of executive function, binding complexity, and memory load on memory performance in individuals with OCD, providing deeper insights that might be missed by traditional meta-regression techniques [38,43].

To test our EBL system, we first needed to develop a scoring system that was both straightforward and capable of generating meaningful ordinal differences across the three EBL dimensions. For instance, a task with a high executive function score should noticeably and practically differ from a task with a lower score on this dimension [90]. Examples include the following: (a) *High*: Tasks requiring advanced executive functions, such as organisational strategies or dual-task demands. (b) *Medium*: Tasks that mix a simple task with a component distracting executive function from the main task, like a delayed match-to-sample (DMTS) task with distractor stimuli. (c) *Low*: Tasks where executive function is used for simple maintenance of information in working memory, such as digit span. We ranked each task on each EBL dimension as high (3), medium (2), or low (1) demand. Additionally, we calculated the total EBL score for each task by summing the scores for each of the EBL dimensions. This allowed us to evaluate the overall effect of the EBL model on memory performance between individuals with OCD and control groups.

By employing this coding method, we were able to investigate the primary impact of the EBL taxonomy simultaneously across multiple domains of memory such as visual and verbal and tasks (e.g., delayed match-to-sample paradigm, reproduction of complex visual shapes, span sequence, spatial span, recall of complex verbal information, recognition memory, and declarative and implicit memory). Additionally, we examined the relative influence of individual moderators and determined which factors of the EBL accounted for the highest degree of variance in memory performance among individuals with OCD. We argued that this approach addressed a limitation previously noted in meta-analyses of memory performance in OCD, where “the classification of individual tasks was not based on reliable criteria” [91].

For example, it enabled us to examine the extent that task demands (e.g., as coded: 1 = low, 2 = moderate, 3 = high) for the total model (total EBL score) and individual moderators (i.e., executive functioning vs. binding complexity vs. load) predicted the memory performance of individuals with OCD (see Figure 2 from [38]). We observed that the overall EBL model predicted memory performance. Specifically, as demand on EBL increased within a given memory task, individuals with OCD exhibited progressively poorer performance. Importantly, we found that executive functioning emerged as the primary driver of the EBL’s impact on memory performance in OCD, mitigating binding complexity and load. In addition, we also observed that poorer memory of those with OCD on visual compared to verbal tasks was also driven by visual tasks placing a greater demand on executive functioning (see Figure 3 from [38]).

Our method quantified the observation that memory impairment in OCD is primarily linked to executive dysfunction [7,14,21,71]. This underscores the significance of our innovative coding and multilevel approach. Had we solely focused on the traditional visual–verbal distinction, our analysis would have missed the subtle yet substantial impact of executive function. This impact relates to binding complexity and load across various tasks and in distinguishing its contribution to memory performance within visual and verbal memory domains in individuals with OCD. Our method offers researchers a means to explore comprehensive extent models, e.g., computational model [92], reciprocal interaction model [93], multidimensional model [94], cognitive behavioural model [95]. We propose it enables them to examine if a broader model with multiple factors explains variance in effect sizes and then to isolate which of these factors contributes to the largest (or smallest) variances in particular outcomes (e.g., executive impairment, memory performance, symptom severity).

## 4. Executive Functioning and Memory Performance in OCD: A Second More Refined Multidimensional Meta-Analysis

After establishing the central role of executive functioning in the memory performance of individuals with OCD, we proceeded to conduct a detailed analysis of the contribution of specific facets of executive control [39]. Using a multilevel coding and multidimensional meta-analytic approach, we investigated and separately analysed the contributions of top-down and bottom-up factors to memory performance in individuals with OCD. This approach was further justified by Fradkin et al. [40], who emphasised the empirical importance of OCD meta-analytic researchers deriving scores consistently from the same sessions, participants, or tasks “when reviewing neuropsychological and cognitive deficits [and that] multilevel meta-analysis… allow[s] the integration of effects of complex structures” (p. 497).

From a top-down perspective and in alignment with our previous conceptualisations of executive function [43], we drew upon Diamond’s Diamond [58,59] (2013, 2020) tripartite taxonomy of executive WM function; for similar conceptualisations of the executive function, see [60,96,97]. This taxonomy includes (a) inhibition, which involves selectively activating task-relevant representations and inhibiting task-irrelevant stimuli and responses; (b) maintenance and updating, encompassing the retention of simple stimuli or features of objects and internal manipulations, as well as the complex updating of representations in WM [60,98,99]; and (c) flexibility/planning, which entails a hierarchically organised integrative system of processing steps driven by executive function to optimise memory performance [100]. Importantly, research has identified each of these domains—specifically attentional control [15,16,76,101,102], maintenance and updating [71,73], and flexibility/planning [15,73,103,104]—as influential factors in the memory functioning of individuals with OCD.

Bottom-up processing explains how a salient external stimulus (e.g., loud noise, personal name, objects related to specific OCD symptoms) captures attention either intentionally or unintentionally [58]. Such stimuli have the ability to automatically draw attention, direct focus, and require the integration and encoding of their features across different sensory modalities [105] and within WM [106]. Indeed, Awh et al. [106] noted that the quality of these initial perceptual inputs across sensory modalities acts as a potential gatekeeper for working memory. Therefore, interference at this early stage inevitably reduces the quality, accuracy, and duration of inputs subsequently held in working memory [107]. To differentiate these processes from active top-down operations, we examined bottom-up processing in terms of the content and characteristics of stimuli, focusing on perceptual integration and perceptual salience. Perceptual integration involves combining fragmentary inputs from different sensory modalities into coherent and organised perceptual objects [108]. Research on perceptual integration impairments in OCD has produced mixed results. For instance, Harting and Markowitsch [109] proposed that difficulties in processing complex visual stimuli (e.g., Rey complex figure task) in OCD may stem from issues with Gestalt perception. In contrast, Moritz & Wendt [110] found no deficits in early perceptual encoding of local elements in individuals with OCD. Perceptual salience refers to the extent to which stimuli attract attention based on their inherent properties, such as brightness, complexity, motion, or emotional impact across modalities, e.g., bright colours, complex or moving stimuli, or acute sounds and feedback [111]. Foa et al. [112] observed that individuals with OC symptoms exhibit perceptual distractibility, perceiving task-irrelevant background noise as louder compared to controls.

Consistent with our initial meta-analysis approach, we standardised each task across dimensions of attentional control, maintenance and updating, flexibility/planning, perceptual integration, and perceptual salience. This system was designed to be straightforward, aiding in replication, and to generate meaningful ordinal distinctions for each dimension. For instance, a task scoring high in maintenance and updating differed significantly in practical ways from one scoring lower on this dimension. Each dimension was defined by specific primary characteristics, and tasks were ranked accordingly: high (3), medium (2), or low (1) demand. For example, maintenance and updating involve actively retaining task-relevant information within working memory (WM) and updating it with more relevant information when necessary, as described in the unity diversity model of executive functions by Friedman et al. [113], Miyake et al. [114], and Miyake & Shah [115]. We then ranked each task ordinally for the dimension of maintenance and updating as follows: *High* (3): Memory tasks that impose significant demands on maintenance capacity, such as reproducing complex visuospatial information (e.g., Rey complex figure test, RCFT), or tasks requiring frequent updating of working memory contents, such as high-load n-back tasks. *Medium* (2): Tasks that moderately challenge maintenance capabilities with limited or no requirement for updating, such as digit span tasks. *Low* (1): Tasks that impose minimal to no demands on the subcomponents, such as simple maintenance well within capacity limits in a delayed matching-to-sample (DMTS) task.

**Figure 5:**
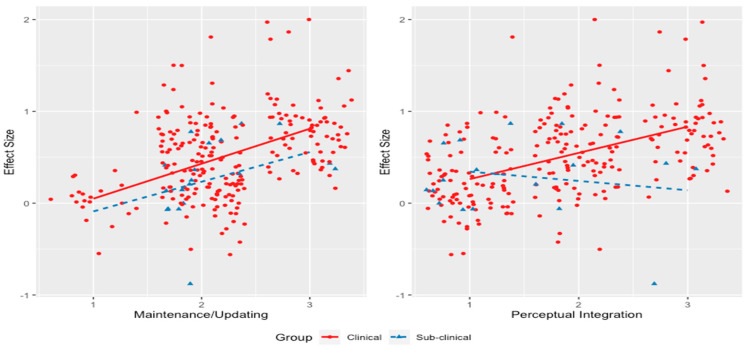
Framework moderation plots for clinical and subclinical groups for maintenance and updating (**top-down**) and perceptual integration (**bottom-up**) [39]. ***Notes.*** The left and right images show the magnitude (effect size) of memory impairment in clinical (solid line) and subclinical (dashed line) OCD participants for maintenance/updating and perceptual integration, respectively. The data indicate that memory impairment is more pronounced in individuals with clinical OCD compared to those with subclinical OCD. Notably, the right image shows an intriguing interaction for perceptual integration: at low levels of perceptual integration (1), no significant differences in memory performance are observed between clinical and subclinical participants, but a clear difference appears at high levels (3).

We observed that as top-down demands increased, individuals with OCD exhibited poorer memory relative to controls. Within the top-down model, only maintenance and updating (compared to attentional control and flexibility/planning) predicted memory performance in OCD (refer to Figure 4, left image). Therefore, within the realm of top-down functions, whether a task involves high maintenance and updating appears to be the primary factor influencing memory differences between individuals with OCD and controls. Similarly, our bottom-up model also predicted memory performance in OCD, showing that as bottom-up demands increased, individuals with OCD had poorer memory relative to controls. Within the bottom-up model, only perceptual integration (compared to perceptual salience) predicted memory performance in OCD (refer to Figure 4, right image). Crucially, analysing these dimensions within the top-down and bottom-up frameworks revealed a lack of significant contribution from the visual–verbal distinction, aligning with findings from our previous research [38]. Specifically, executive function similarly showed nonsignificant differences between tasks of a visual or verbal nature. Furthermore, when comparing clinical and subclinical OCD participants, we discovered that maintenance and updating (top-down; refer to Figure 5, left image) and perceptual integration (bottom-up; refer to Figure 5, right image) were the only significant predictors of memory performance in the clinical OCD group but not in the subclinical group.

In summary, our findings emphasise the crucial role of executive functioning in memory performance among individuals with OCD. Through a detailed analysis of top-down and bottom-up processes, we uncover the distinct contributions of attentional control, maintenance and updating, flexibility/planning, perceptual integration, and perceptual salience to memory outcomes. Notably, maintenance and updating emerge as key predictors within the top-down framework, while perceptual integration significantly impacts memory performance in the bottom-up model. These findings offer valuable insights into the intricate interplay between cognitive processes and memory deficits in OCD, which allowed us to identify novel targets for interventions targeting specific executive functions to enhance clinical outcomes.

## 5. Enhancing Clinical Relevance: Insights from a Multimodal Meta-Analysis Perspective

We propose that by building upon our refined understanding of executive functioning’s role in memory performance among individuals with OCD, we now shift our focus towards enhancing clinical relevance through a multimodal meta-analysis perspective. By integrating our findings within the broader context of existing research and utilizing a multimodal meta-analysis lens, we aim to delve deeper into the cognitive processes underlying OCD and pave the way for more effective interventions.

Indeed, we utilised our findings from Harkin et al. [39] to formulate a potential explanation for memory impairment in OCD within the framework of our bottom-up and top-down approach. This explanation is clarified by faulty gating mechanisms within early sensory processing [116,117] and WM [118,119]. Sensory gating refers to the process of filtering out task-irrelevant stimuli from the external environment [120], influenced by factors such as anxiety and selective attention [121]. Deficits in sensory gating are present in several mental illnesses and contribute to cognitive disturbances [122], with impairments in sensory gating of early perceptual stimuli observed in OCD [123,124]. Theories of maintenance and updating propose that efficient WM relies on a “gating” mechanism to manage changing inputs and task demands; for reviews, see [118,119,125]. A closed gate helps maintain relevant information within WM by keeping irrelevant information out, thus protecting capacity limits [126]. Conversely, an open gate facilitates updating by removing, replacing, or adding new information to meet evolving task demands [127].

**Figure 6:**
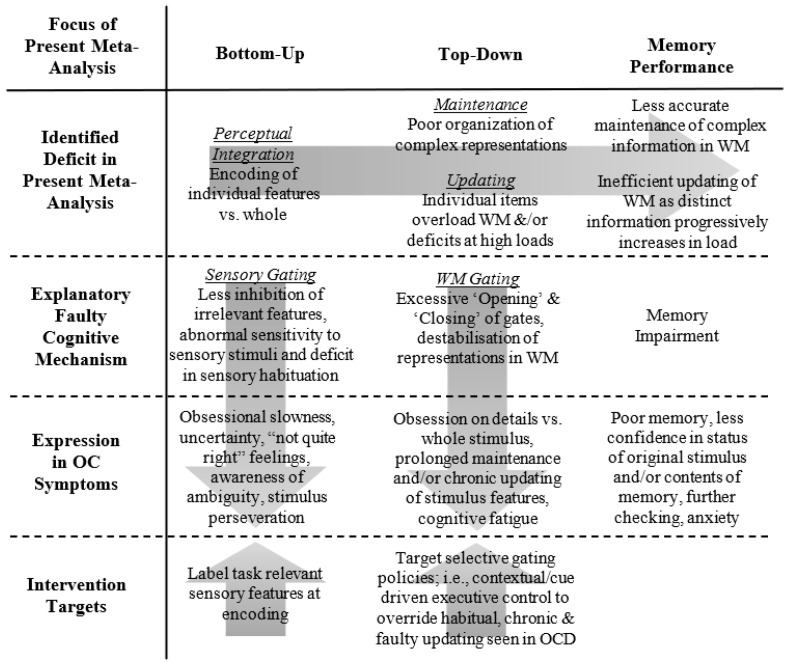
Overview of present meta-analysis, faulty cognitive mechanisms, expression in OC symptoms, and interventions [39]. ***Notes.*** In this figure, arrows depict proposed relationships and/or directions of potential causality within each of the four main sections. (a) *Identified Deficits in Present Meta-Analysis*: The arrow illustrates how impairments in perceptual integration influence the maintenance and updating of information in working memory (WM) and subsequently impact overall memory performance in individuals with OCD. (b) *Exploratory Faulty Cognitive Mechanism and Expression in OC Symptoms*: The arrows indicate the direction of our proposed faulty gating mechanisms (sensory and WM) and their manifestation in OCD symptoms. (c) *Intervention Targets*: The arrow in this context shows how the proposed intervention could potentially address issues in OCD symptoms and the identified faulty gating mechanisms.

Specifically, within OCD, we propose that the overloading and overuse of this ‘gating’ mechanism may account for the pattern of memory impairments observed in our meta-analysis and potentially contribute to clinical OC symptoms. In Figure 6, we summarise the relationship between the deficits identified in our meta-analysis, their potential effects on memory performance in OCD, the faulty gating mechanisms implicated, their manifestation in OC symptoms, and potential targets for intervention. Individuals with OCD experience deficits in early perceptual integration, leading them to focus on and encode individual pieces of information rather than the whole. Consistent with our data pattern, this tendency occurs regardless of whether a task is visual or verbal in nature and seems to be influenced by the overall complexity of the stimuli used in a given memory task [38]. As a result, a series of independent and potentially disparate items enter WM [106], leading to the excessive opening and closing of the gate to update and maintain this information in WM. This suggests a shift from a global gating mechanism to one that is highly selective, stimulus-driven, and retroactive in nature [119,125,128]. Overloading this gating mechanism likely destabilises the accuracy of the information maintained and updated within WM; for a review, see [129]. This assertion aligns with the observation that individuals with OCD experience difficulties in tasks that necessitate explicit updating (e.g., span sequence, n-back) to achieve accurate working memory (WM) performance, e.g., [72].

Finally, our gating explanation sheds light on the differences between clinical and subclinical patterns observed across the bottom-up and top-down frameworks. Specifically, while individuals with clinical OCD showed more pronounced impairments in memory compared to those with subclinical OCD (e.g., *d* = 0.51 versus 0.30, respectively), perceptual integration (bottom-up) and maintenance and updating (top-down) only predicted poor memory performance in the clinical group. Thus, while memory impairment is evident across both subclinical and clinical OCD groups, these two dimensions exacerbate memory deficits specifically at the clinical level. This observation aligns with the symptoms experienced by individuals with OCD, highlighting the range of impairments and challenges they face related to stimuli specific to their symptoms [130], for example, as shown in Figure 6, poor memory [70], lack of confidence [131], a desire to physically check and recheck [132], implicit awareness of ambiguity [133], cognitive fatigue [134], and anxiety and avoidance [135].

## 6. Bridging Research and Practice: Insights from a Multimodal Meta-Analysis Perspective

### 6.1. Targeted Interventions for Cognitive Dysfunction in OCD: Bridging Theory to Practice

Using our findings as a foundation, we propose intervention to address potential faulty cognitive mechanisms we identified at a sensory and WM gating level. As such, we aim to address Ouimet et al.’s [44] critique of cognitive research in OCD. They argue that to positively impact therapeutic outcomes, we must move beyond a simplistic explanation of cognitive processes in OCD.

### 6.2. Labelling—An Intervention in Faulty Sensory Gating

A possible intervention in faulty sensory gating is an established experimental manipulation of bottom-up processing called verbal labelling [136]. This approach associates a complex visuospatial representation with a verbal label, e.g., polygons with colours [137], which improves the early perceptual encoding of the stimulus and subsequent memory performance [138]. More specifically, task-relevant verbalisations improved, i.e., directed attention to relevant stimuli [139], whereas unrelated labelling reduced task performance [140] and also increased the speed of switching between tasks [141]. Furthermore, labelling (and simple pointing) is an effective means to improve relevant stimuli selection in those where this cognitive facility is not fully developed [139], a discovery that parallels what we observed in the memory performance of individuals with OCD in the current review. We suggest that labelling may serve to target faulty sensory gating (i.e., focusing on irrelevant features, sensory habituation) and associated OC symptoms (i.e., obsessional slowness) (see Figure 6). Simply within a given memory task or idiographic context of OC symptom provocation, those with OCD could be asked to verbally label (internally or aloud) task-relevant features relevant to accurate task performance. In an applied context, labelling could take the form of targeting attention in early encoding for aspects of an image that are task-relevant and then measuring subsequent memory performance and OC symptoms (e.g., desire to check and recheck the original stimulus).

### 6.3. Input-Gating Policies—An Intervention in Faulty WM Gating

As a solution to faulty gating in WM, we draw upon research that indicates gating mechanisms within WM are sensitive to manipulation as they operate according to gating policies [125,142]. In the first instance, a selective gating mechanism promotes the maintenance and updating of information in WM via input gates that open and close, respectively [118]. Within this, optimal WM performance requires a gating policy of when to open and close the gate in accordance with the demands of a task [143]. Bhandari and Badre [142] manipulated gating policies within a WM task. They showed that the selection of a given gating policy (e.g., open versus closed) was determined by the contextual demands (e.g., via a cue) of a task and transferred over to subsequent trials [144]. They reported that repetitively requiring participants to adopt a selective input-gating policy encouraged the proactive and efficient encoding of stimulus inputs into WM. Of relevance to the present findings on maintenance and updating in OCD, they suggested that such a process reduces load within WM (i.e., efficient binding of complex information) and interference from irrelevant stimuli (e.g., prolonged updating of individual stimulus features; see Figure 6). We propose that while faulty gating policies show a degree of perseveration consistent with symptoms of OCD, they are fortunately malleable to intervention.

We suggest that targeting input-gating policies in WM may serve as an intervention to what we proposed in terms of faulty WM gating and OC symptoms, i.e., excessive opening and closing of gates in WM leading to chronic maintenance and updating of isolated features, destabilisation of WM, and cognitive fatigue (see Figure 6). As such, we propose an intervention similar to attentional bias modification tasks (for a review, see [145]) that are already employed to treat those with OCD [146], specifically, training those with OCD to adopt a selective input-gating policy, one that encourages the selective entry of information into WM, via advanced preparation. While targeting gating policies in this manner may seem somewhat removed from OC symptoms per se, research indicates that training cognitive control using this approach improves executive control performance in novel untrained contexts [144]. It also lends itself to a simple method of delivery via mobile devices (apps) either before or during symptom provocation [147]. Therefore, we infer that a similar transfer from a narrow cognitive intervention to a more specific context that is problematic for those with OCD is possible, for example, from performance on higher loads of the *n*-back task to turning off the stove and not returning. 

Evidence from neuroscience shows that the delivery of transcranial magnetic stimulation (TMS) to the left dorsolateral prefrontal cortex (DLPFC) improves performance on the *n*-back task [148]. They concluded that stimulation of the left DLFC improved maintenance (i.e., enhanced capacity via improved distractor suppression) and updating (i.e., using contextual cues or task-related demands) in spatial WM [149]. We suggest this indicates a possible therapeutic link between TMS and potential targeting of WM gating mechanisms (open and closing, gating policies) in OCD patients who suffer from established impairments in inhibitory function, encoding, and WM performance. Future research needs to validate the strength of these latter assertions.

Therefore, future research in OCD should continue to explore the intricate relationship between cognitive processes, such as sensory gating and working memory, and the manifestation of symptoms. Specifically, further investigation into how these cognitive deficits contribute to specific symptom domains within OCD, such as intrusive thoughts [150,151] or compulsive behaviours [152], could provide valuable insights into the underlying mechanisms of the disorder. For example, the cognitive profile of checkers versus washers has been shown to be different [15], with impairments in inhibition associated with poorer memory in checkers but not washers, e.g., [76]. An mi-MA could provide valuable insights into why inhibition is specifically associated with poorer memory in individuals who exhibit checker-like behaviours compared to washers. By simultaneously examining various cognitive domains beyond inhibition, such as attentional control, e.g., [153], working memory, e.g., [67], and cognitive flexibility, e.g., [154], researchers could elucidate potential interactions or mediating factors that contribute to this association. For instance, it is possible that individuals with checker-like behaviours not only demonstrate deficits in inhibition but also exhibit impairments in other cognitive functions that are crucial for memory encoding, storage, or retrieval. Moreover, considering individual differences within each cognitive domain, such as variances in the severity of OCD symptoms or levels of comorbidities, employing the mi-MA approach could aid in pinpointing factors that intensify the impact of inhibition and memory in individuals with checking-related OCD.

Similarly, research suggests that inhibitory control deficits may not be a universal feature across various disorders. While previous studies proposed that inhibitory impairments are transdiagnostic [97], recent findings challenge this view. Mirabella [155] reviewed studies on several psychiatric disorders, including OCD, Tourette syndrome (TS), attention-deficit/hyperactivity disorder (ADHD), autism spectrum disorder (ASD), and primary motor stereotypies (pMS), and found striking differences in motor and response inhibitory function across these conditions. At its core, motor inhibition is the ability to suppress a planned or already initiated action [156]. More specifically, response inhibition has at least two neuropsychological domains: (1) reactive inhibition, i.e., the ability to stop a response outright when a stop instruction is presented, and (2) proactive inhibition, i.e., the ability to adapt the motor strategy according to the context where an individual is embedded and to previous knowledge.

For instance, drug-naïve TS patients did not exhibit impairments in inhibitory control [157]. In contrast, both reactive and proactive inhibitory domains are severely compromised in OCD [157]. ADHD and pMS show selective impairments in reactive inhibition [158,159], whereas ASD is characterised by deficiencies in proactive inhibitory control [160]. Importantly, Mirabella et al. [155] also identified a link between specific deficits in inhibitory control subdomains and the clinical manifestations of these disorders.

Overall, this evidence suggests that the mechanisms underlying the inability to control urges are extremely heterogeneous and cannot be attributed to a general impairment of motor inhibition. Therefore, recent findings do not support the hypothesis that inhibitory deficits represent a transdiagnostic feature of neurodevelopmental disorders with poor impulse control. Specifically, Mirabella et al. [158] observed that OCD patients exhibit significant grey matter (GM) volume reductions in areas such as the middle frontal gyrus, inferior frontal gyrus, medial orbitofrontal gyrus, mid-cingulate gyrus, and insula. These reductions were inversely correlated with measures of reactive inhibition (e.g., stop-signal reaction time; SSRT) and the severity of OCD symptoms.

Given these findings, we propose that the mi-MA approach may facilitate the investigation of the interaction between core aspects of executive function (e.g., inhibition, maintenance and updating, set-shifting), GM volume reductions, and OCD symptom severity. In addition, it may offer a means to examine the likely differing contributions of different aspects of inhibitory control (i.e., motor, reactive, proactive) to symptom development and severity in OCD and other disorders such as TS, ADHD, ASD, and pMS. As such, it may help identify specific and optimal targets of inhibitory function for each disorder, which can be addressed in treatment, e.g., within attention bias modification interventions [161,162,163], to enhance outcome effectiveness. Lastly, the mi-MA approach could be instrumental in exploring the specific nature of inhibitory control deficits and their neural correlates across different disorders. This highlights a valuable avenue for the mi-MA approach to investigate cognitive function and its neural underpinnings both within OCD and across various psychiatric conditions.

Indeed, this highlights a core benefit of the mi-MA approach, in its ability to allow researchers to extract and quantify multiple measures from the same group of participants for a given outcome. Examples include aspects of cognition, e.g., thought–action fusion, executive–memory relationship, intolerance of uncertainty, confidence in memory [12,43,164,165]; subsymptoms, e.g., checking, washing [15]; comorbidities, e.g., depression, anxiety [166]; medication status, e.g., naive, present, absent, total dosage [167]; treatment type, e.g., medicated, cognitive behavioural therapy, combination [168,169]; and neural correlates, e.g., Uhre et al. [170,171,172]. By integrating and analysing multiple measures simultaneously, researchers can gain a deeper insight into the complex interplay of factors contributing to the phenomenon under investigation, leading to more robust and detailed conclusions.

Additionally, longitudinal studies tracking cognitive functioning over time and in response to treatment interventions could shed light on the dynamic nature of cognitive processes in OCD and their role in treatment outcomes. Furthermore, there is a need for research that examines the effectiveness of interventions targeting these cognitive deficits, such as cognitive remediation or attention training, in improving overall functioning and reducing symptom severity in individuals with OCD. Overall, future research endeavours should aim to bridge the gap between theoretical models of OCD and clinical practice by translating findings into actionable interventions that improve the lives of individuals affected by the disorder.

### 6.4. Beyond Efficacy: Targets and Mechanisms of OCD Interventions Using mi-MA

A critique of intervention research in the context of OCD was raised by Himle et al. [173] who noted a prevailing overemphasis on efficacy evaluations, primarily through randomised controlled trials (RCTs), normally comparing cognitive behaviour therapies (CBTs) to alternative modalities, such as exposure and response prevention (EX/RP) [174], behavioural activation [175], and combinations of other interventions, e.g., selective serotonin reuptake inhibitors (SSRIs) [169]. However, such studies often rely on ad hoc assumptions regarding underlying mechanisms. Himle [173] observed an “inadequate understanding of their actual mechanism(s) of action prohibits us from knowing how variations in techniques … might activate or interfere with the purported learning process and thus desired outcomes” (p. 188). It is an important caveat that while studies claim to utilise CBT/EX/RP, the actual execution of these therapies can significantly vary, with some demonstrating minimal procedural congruence [173].

Given the significant implications at hand, there exists an urgent necessity to explore further the landscape of interventions for OCD, particularly CBT, which is widely acclaimed as the “gold-standard” treatment for this condition [22,176,177,178]. Indeed, while numerous RCTs, reviews, and meta-analyses have confirmed the effectiveness of CBT in alleviating OCD symptoms [19,26,179], few, if any, have systematically examined the specific facets and mechanisms of CBT that underlie this success. Most meta-analyses compare CBT to an comparator condition (e.g., pharmacological or control), aggregate effect sizes across a set of studies, and then conclude on the effectiveness of CBT in treating OCD [26,180,181].

To bridge this critical knowledge gap, we propose a shift in focus for future research using the mi-MA approach. Specifically, using the mi-MA approach to dissect facets of CBT, along with associated intervention combinations like exposure and response prevention (EX/RP) [182] and selective serotonin reuptake inhibitors (SSRIs) [183]. In this context, the mi-MA approach serves three potential purposes. First, it involves comparing overall effect sizes for CBT with other interventions (e.g., rational emotive therapy; RET), combinations (e.g., CBT + SSRIs), and controls (e.g., active, passive, wait-list control) as typically undertaken in classic meta-analysis. Engaging in this process will function as a reality check, enabling researchers to juxtapose results with those of other meta-analyses and subsequently pinpoint the facets of interventions that yield the most favourable therapeutic outcomes.

Using this information, it is then possible to delve deeper into the identification of specific elements within cognitive, behavioural, and pharmacological interventions. These elements may include cognitive techniques (e.g., identifying and challenging irrational OC thoughts, using thought records, targeting thought–action fusion, imaginal exposure), behavioural strategies (e.g., in vivo exposure, ritual prevention), and pharmacological factors (e.g., medication type, dosage, prescribed versus not). Such an approach, with the mi-MA paradigm, will allow researchers to quantify their respective contributions to therapeutic outcomes for individuals with OCD.

Moreover, it is common to observe varying outcomes in interventions for OCD across different modalities. For example, Ost et al. [184] found that CBT (70%) and combined CBT and SSRIs (66%) had significantly better outcomes than SSRIs alone (49%), the placebo (29%), and the waitlist control (13%). In a large-scale study, Foa et al. [185] reported complete and treated response rates for EX/PR and clomipramine (i.e., a serotonin reuptake inhibitor; SRI) of 79% and 70%, EX/RP in isolation was 86% and 62%, clomipramine alone was 48% and 42%, and 10% and 8% for the placebo. In addition, remission of OC symptoms following CBT and/or SSRIs presents a mixed picture, with up to 50% of patients experiencing a relapse to their previous OC symptom levels [186,187,188]. Ost et al. [184] reported higher CBT remission rates (53%) than isolated SSRI intervention (24%). Worryingly, research has also indicated that 78.6% of patients experience a remission of symptoms after a 2-year course of CBT [189]. It has also been noted that after receiving the recommended dosage of CBT, many individuals with OCD continue to experience chronic residual symptoms after intervention [173]. Further complicating this is the finding that those with OCD and comorbid depression experience less positive outcomes with CBT [175].

Thus, considering these points, the mi-MA approach offers a means to investigate the following pressing research questions. (a) What factors contribute to the similar intervention outcomes between CBT alone and the combination of CBT and SSRIs, despite SSRIs alone having lower outcomes? (b) What factors contribute to the variation in intervention outcomes for OCD across different intervention modalities, such as CBT, SSRIs, EX/RP, and placebo? (c) What differences exist between different pharmacological interventions (e.g., SSRIs and SRIs) when combined with different modalities of intervention, e.g., CBT and intensive EX/RP? (d) What factors influence relapse and remission rates, and how are these influenced by factors such as intervention modality, intervention duration, and comorbid depression in OCD patients?

In summary, given these disparities, open research questions, and lack of a specific understanding of the mechanisms of action (see [173]), we propose that the mi-MA approach offers a means to pinpoint pivotal factors inherent to interventions, such as specific elements and quantities of CBT and pharmacological components. This method also allows for the examination of comparative aspects, like CBT versus SSRIs versus combined therapy, which can be harnessed to enhance outcomes for OCD. We believe it will enrich the therapeutic landscape by improving the research available to therapists and therein the effectiveness of interventions available to those with OCD. We hope that we have demonstrated that the mi-MA approach provides a means for researchers to explore novel aspects and combinations of interventions, as well as identify effective intervention–outcome mechanism relationships derived from the meta-analysis.
